# Examination at Mt. Airy, N. C.

**Published:** 1874-03

**Authors:** 


A Short and Concise Account of Eliza and Mary Chulkhurst—Who were born joined together by the hips and shoulders, in the year of our Lord, 1100, at Biddenden, in the county of Kent, commonly called the Biddenden Maids. They lived together in the above state thirty-four years, at the expiration of which time one of them was taken ill and in a short time died; the surviving one was advised to be separated from the body of her deceased sister by dissection, but she absolutely refused the separation by saying these words,—"As we came together we will also go together," and in the space of about six hours after her sister's decease she was taken ill and died also.

By their will they bequeathed to the churchwardens of the parish of Biddenden and their successors churchwardens for ever, certain pieces or parcels of land in the parish of Biddenden, containing twenty acres, more or less, which now let at 40 guineas per annum. There are usually made in commemoration of these wonderful phenomena of nature, about 1,000 rolls with their impressions printed on them, and given away to all strangers on Easter Sunday after divine service in the afternoon; also about 300 quartern loaves and cheese in proportion, to all the poor inhabitants of the said parish.

